# Identification of serum biomarkers linking myocardial fibrosis, systolic dysfunction and outcomes in patients with severe aortic stenosis

**DOI:** 10.1093/cvr/cvag073

**Published:** 2026-03-30

**Authors:** Svante Gersch, João P Ferreira, Andreas Leha, Philipp Bengel, Stephan von Haehling, Andreas Fischer, Bo E Beuthner, Elisabeth M Zeisberg, Miriam Puls, Constanze Schmidt, Karl Toischer, Gerd Hasenfuß, Moritz Schnelle

**Affiliations:** Department of Cardiology and Pneumology, University Medical Center Göttingen, Robert-Koch-Str. 40, 37075 Göttingen, Germany; German Centre for Cardiovascular Research (DZHK), partner Site Lower Saxony, Robert-Koch-Str. 40, 37075 Göttingen, Germany; Department of Surgery and Physiology, Cardiovascular Research and Development Center, Faculty of Medicine, University of Porto, Alameda Prof. Hernâni Monteiro, 4200-319 Porto, Portugal; Department of Medical Statistics, University Medical Center Göttingen, Humboldtallee 32, 37073 Göttingen, Germany; Department of Cardiology and Pneumology, University Medical Center Göttingen, Robert-Koch-Str. 40, 37075 Göttingen, Germany; German Centre for Cardiovascular Research (DZHK), partner Site Lower Saxony, Robert-Koch-Str. 40, 37075 Göttingen, Germany; Department of Cardiology and Angiology, Justus-Liebig-University, Klinikstrasse 33, 35390 Giessen, Germany; Department of Cardiology and Pneumology, University Medical Center Göttingen, Robert-Koch-Str. 40, 37075 Göttingen, Germany; German Centre for Cardiovascular Research (DZHK), partner Site Lower Saxony, Robert-Koch-Str. 40, 37075 Göttingen, Germany; German Centre for Cardiovascular Research (DZHK), partner Site Lower Saxony, Robert-Koch-Str. 40, 37075 Göttingen, Germany; Department of Clinical Chemistry, University Medical Center Göttingen, Robert-Koch-Str. 40, 37075 Göttingen, Germany; Institute of Clinical Chemistry, Medical Faculty Mannheim of Heidelberg University, Theodor-Kutzer-Ufer 1-3, 68167 Mannheim, Germany; Department of Cardiology and Pneumology, University Medical Center Göttingen, Robert-Koch-Str. 40, 37075 Göttingen, Germany; German Centre for Cardiovascular Research (DZHK), partner Site Lower Saxony, Robert-Koch-Str. 40, 37075 Göttingen, Germany; Department of Cardiology and Pneumology, University Medical Center Göttingen, Robert-Koch-Str. 40, 37075 Göttingen, Germany; German Centre for Cardiovascular Research (DZHK), partner Site Lower Saxony, Robert-Koch-Str. 40, 37075 Göttingen, Germany; Department of Cardiology and Pneumology, University Medical Center Göttingen, Robert-Koch-Str. 40, 37075 Göttingen, Germany; German Centre for Cardiovascular Research (DZHK), partner Site Lower Saxony, Robert-Koch-Str. 40, 37075 Göttingen, Germany; Department of Cardiology and Pneumology, University Medical Center Göttingen, Robert-Koch-Str. 40, 37075 Göttingen, Germany; German Centre for Cardiovascular Research (DZHK), partner Site Lower Saxony, Robert-Koch-Str. 40, 37075 Göttingen, Germany; Department of Cardiology and Pneumology, University Medical Center Göttingen, Robert-Koch-Str. 40, 37075 Göttingen, Germany; German Centre for Cardiovascular Research (DZHK), partner Site Lower Saxony, Robert-Koch-Str. 40, 37075 Göttingen, Germany; Department of Cardiology and Pneumology, University Medical Center Göttingen, Robert-Koch-Str. 40, 37075 Göttingen, Germany; German Centre for Cardiovascular Research (DZHK), partner Site Lower Saxony, Robert-Koch-Str. 40, 37075 Göttingen, Germany; German Centre for Cardiovascular Research (DZHK), partner Site Lower Saxony, Robert-Koch-Str. 40, 37075 Göttingen, Germany; Department of Clinical Chemistry, University Medical Center Göttingen, Robert-Koch-Str. 40, 37075 Göttingen, Germany

**Time of primary review: 57 days**

Aortic valve stenosis (AS) is the most prevalent acquired heart valve disease in the Western world, affecting over 2% of individuals over the age of 60 and imposing a significant burden of morbidity and mortality.^1^ Transcatheter aortic valve replacement (TAVR) has become the preferred choice of intervention in the management of aortic valve disease, with a notable increase in the number of procedures performed over the last years due to an aging society.^2^ Consequently, effective risk stratification becomes essential for selecting suitable candidates and improving outcomes in severe AS. Pathophysiologically, the progression of AS affects the left ventricle, leading to myocardial adaptations such as cardiac hypertrophy and diffuse myocardial fibrosis (MF).^3^ Emerging evidence suggests a link between the degree of MF and impaired outcomes after AVR in severe AS patients.^4^ While cardiac biopsy remains the most reliable method for measuring MF, it is an invasive procedure that poses significant risks of severe complications. Therefore, there is a pressing need for simpler methods to assess MF levels.^5^ Approaches to non-invasively identify serum biomarkers to estimate the degree of MF in patients with AS remain scarce.^5^ To address this issue, we conducted a comprehensive proteomic analysis, utilizing a large panel of serum biomarkers. Specifically, we analyzed 184 biomarkers using the Olink® technology (CVDII and CVDIII panels; Olink®, Uppsala, Sweden) in 169 serum samples from our well-characterized TAVR cohort,^4^ collected prior to the procedure, and examined their associations with histologically assessed MF, systolic dysfunction and cardiovascular mortality.

Firstly, the cohort was dichotomized based on the median value of histologically assessed MF (measured as collagen volume fraction in myocardial tissue samples obtained during TAVR, available for *n* = 100), and secondly according to the echocardiographic Simpson’s biplan method obtained left ventricular ejection fraction (LVEF) above or below 50%. For MF assessment, endomyocardial LV biopsies were obtained from the basal anteroseptum immediately after transcatheter valve deployment, fixed in paraformaldehyde, paraffin-embedded, sectioned (3 μm), and stained with Masson’s trichrome. MF was quantified as the proportion of blue-stained area relative to total tissue area by two independent, clinically blinded observers using quantitative morphometry. Associations between serum biomarkers and MF as well as systolic function, i.e. LVEF, were studied using logistic regression models, adjusted for clinically relevant parameters age, sex and renal function (via estimated glomerular filtration rate, eGFR, using the CKD-EPI formula). The resulting model coefficients (in the form of odds-ratios, OR) are reported with 95% confidence intervals and *P*-values testing the null hypothesis of no association. Survival analysis was performed by means of Cox proportional hazard models for the cause-specific hazard of cardiovascular death adjusting for age, sex, renal function and LVEF. All tests were two-sided, and *P*-values <0.05 were considered statistically significant and were not adjusted for multiple testing due to the exploratory nature of the study.

Compared with MF− (MF below the median), study participants belonging to MF + (MF above the median) were younger and more often male (*Figure 1A*). Aside from a higher prevalence of type 2 diabetes, the distribution of cardiovascular comorbidities was similar between groups. Echocardiographically, MF+ showed lower LVEF and a higher frequency of low-flow, low-gradient AS, a haemodynamic subtype characterized by reduced LVEF with lower peak aortic jet velocity and transvalvular gradients (*Figure 1A*). MF was found to be inversely associated with LVEF (*P* < 0.001) (*Figure 1B*). Further patient details are provided in *Figure 1A*. Regarding the proteomic analysis, several serum biomarkers were found to be significantly associated with the extent of histological MF (*Figure 1C*). In this analysis, among others, higher levels of Brain natriuretic peptide (BNP) [OR 1.36 (1.09–1.73), *P* = 0.009], N-terminal prohormone of BNP (NT-proBNP) [OR 1.43 (1.10–1.90), *P* = 0.010], Growth hormone (GH) [OR 1.53 (1.15–2.09), *P* = 0.005], and Fibroblast growth factor 23 (FGF23) [OR 1.51 (1.07–2.28), *P* = 0.032] as well as lower levels of Stem cell factor (SCF) [OR 0.40 (0.17–0.86), *P* = 0.027] were significantly associated with a greater extent of MF (i.e. values above the median) (*Figure 1C*). We next assessed whether these biomarkers were also associated with cardiac dysfunction—defined as reduced LVEF—by stratifying the entire cohort (*n* = 169) into patients with preserved (LVEF ≥50%) and reduced systolic function (LVEF <50%). Patient details can be found in *Figure 1D*. In total, 35 biomarkers were significantly linked to reduced LVEF in severe AS (*Figure 1E*). As expected, natriuretic peptides showed strong associations. Notably, several biomarkers were significantly linked with both MF (*Figure 1C*) and systolic dysfunction, including BNP, NT-proBNP, GH, FGF23 and SCF (*Figure 1E*). Finally, we evaluated associations with cardiovascular mortality during a maximum follow-up of 1812 days (median 658 days; interquartile range, 392–1042 days). After adjustment for age, sex, renal function, and LVEF, several biomarkers previously linked to MF and systolic dysfunction (*Figure 1C* and *E*) also carried prognostic significance, i.e. BNP (HR 1.27, 95% CI 1.05–1.54, *P* = 0.013), NT-proBNP (HR 1.37, 95% CI 1.12–1.70, *P* = 0.003), SCF (HR 0.45, 95% CI 0.26–0.79, *P* = 0.005), FGF23 (HR 1.29, 95% CI 1.08–1.56, *P* = 0.006), and GH (HR 1.20, 95% CI 1.01–1.45, *P* = 0.044) (*Figure 1F*).

**Figure 1:**
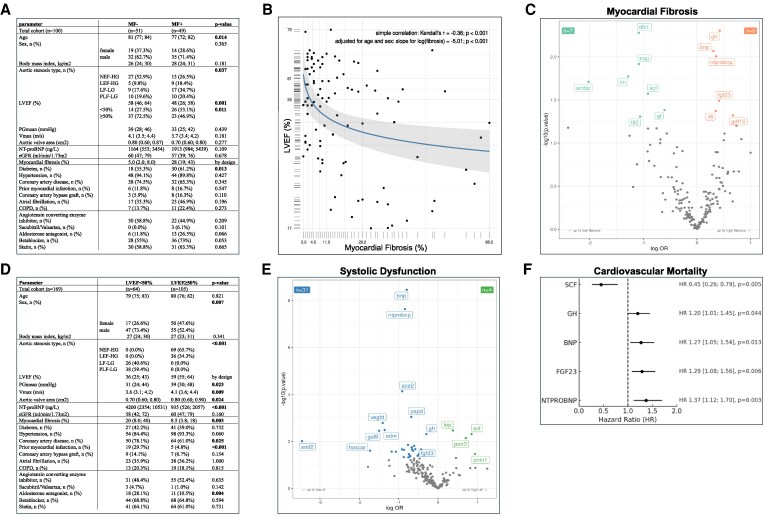
(*A*) Baseline characteristics of the study population dichotomized by median of histologically quantified myocardial fibrosis (MF+/MF−; *n* = 100). Continuous variables are presented as median with Inter-Quartile-Range (IQR). P-values testing the null hypothesis of no group difference are shown. Descriptive two-group comparisons were performed using Boschloo’s exact test, Fisher’s exact test, Welch’s *t*-test, or the Brunner-Munzel test, as appropriate. (*B*) Scatter plot illustrating the association between MF and LVEF, adjusted for age and sex [multivariable linear regression using log(fibrosis)]. Each point represents one participant (*n* = 100); the blue line shows the fitted linear regression with 95% confidence band (grey). (*C*) Volcano plot displaying significant associations between serum biomarker levels and MF levels in the study cohort (*n* = 100) adjusted for age, sex and renal function. Biomarkers labelled in orange are elevated in MF+, those labelled in green lower in MF+. OR and 95% CI and respective *P*-values are shown. (*D*) Baseline characteristics of the study population dichotomized by systolic function (LVEF < 50%/LVEF ≥ 50%; *n* = 169). Continuous variables are presented as median with Inter-Quartile-Range (IQR). P-values testing the null hypothesis of no group difference are shown. Descriptive two-group comparisons were performed using Boschloo’s exact test, Fisher’s exact test, Welch’s *t*-test, or the Brunner-Munzel test, as appropriate. (*E*) Volcano plot displaying significant associations between serum biomarker levels and systolic function, i.e. LVEF < 50% vs. ≥50%, in the study cohort (*n* = 169) adjusted for age, sex and renal function. Biomarkers labelled in blue are elevated when systolic function is reduced, those labelled in green when function is preserved. For clarity, only the 15 most significant biomarkers are displayed. (*F*) Associations between serum biomarkers and cardiovascular mortality following TAVR (*n* = 169). Only proteins with significant associations to both MF and systolic dysfunction, as shown in *Figure 1C* and *E*, are displayed. Survival analysis was conducted using Cox proportional hazard models, adjusted for age, sex, renal function, and LVEF (dichotomized at 50%). Hazard Ratios (HR), 95% CI and respective *P*-values are shown. ACE2, Angiotensin-Converting Enzyme 2; ADM, Adrenomedullin; AMBP, Alpha-1-microglobulin/Bikunin Precursor; BNP, B-type Natriuretic Peptide; CNTN1, Contactin-1; COPD, Chronic Obstructive Pulmonary Disease; DLK1, Delta-like Non-Canonical Notch Ligand 1; eGFR, Estimated Glomerular Filtration Rate; FGF23, Fibroblast Growth Factor 23; GAL9, Galectin-9; GDF15, Growth Differentiation Factor 15; GH, Growth Hormone; GT, Gastrotropin; HOSCAR, Osteoclast-Associated Immunoglobulin-Like Receptor; IGFBP2, Insulin-Like Growth Factor-Binding Protein 2; IL6, Interleukin 6; LEP, Leptin; LF-LG, Low-Flow, Low-Gradient Aortic Stenosis; LVEF, Left Ventricular Ejection Fraction; NF-HG, Normal-Flow, High-Gradient Aortic Stenosis; NTPROBNP, N-terminal prohormone of brain natriuretic peptide; PGmean, Mean Transvalvular Pressure Gradient; PLF-LG, Paradoxical Low-Flow, Low-Gradient Aortic Stenosis; PON3, Paraoxonase 3; PSPD, Pulmonary Surfactant Protein D; SCF, Stem Cell Factor; SOD2, Superoxide Dismutase 2; TGM2, Transglutaminase 2; TLT2, TREM-like Transcript 2 Protein; TM, Thrombomodulin; TNFSF13B, TNF Ligand Superfamily Member 13B; TRAILR2, TNF-Related Apotosis-Inducing Ligand Receptor 2; TRAP, Tartrate-Resistant Acid Phosphatase; VEGFD, Vascular Endothelial Growth Factor D; Vmax, Peak Aortic Jet Velocity.

To the best of our knowledge, this is the first study to identify serum biomarkers linking MF, systolic dysfunction, and cardiovascular mortality in a cohort of AS patients. The observed association between higher MF and lower LVEF is consistent with the concept that progressive structural remodelling contributes to systolic impairment.^4^ Increased MF replaced contractile myocardium and promote adverse ventricular stiffening and altered geometry, thereby reducing effective contractility and systolic reserve. Together, these findings support MF as a marker of remodelling severity with functional consequences for systolic performance. Furthermore, our observations align with prior AS data demonstrating associations between natriuretic peptide levels and fibrotic remodelling in the heart. For instance, NT-proBNP levels were shown to correlate with MRI-derived collagen volume fraction, relating to reduced LVEF and post-TAVR remodelling, and predicting mortality across AS severity.^2,6,7^ Experimental work shows that FGF23 can promote cardiac hypertrophy, MF and thus worsens diastolic function.^8^ Clinically, higher FGF23 predicts incident heart failure with reduced ejection fraction (HFrEF) and may contribute causally to calcific AS. Building on these findings and our observations, we propose that FGF23 contributes to adverse remodelling and clinical deterioration in patients with AS and reduced LVEF. Thus, FGF23 represents a promising circulating biomarker and potential therapeutic target that warrants further investigation. While GH regulates growth and collagen turnover, its cardiac effects are context-dependent; thus, GH can down-modulate transforming growth factor-β (TGF-β) in cardiac fibroblasts, attenuate MF and apoptosis, and improve endothelial function.^9^ Elevated GH levels in our cohort thus may reflect a compensatory response to adverse remodelling. In contrast to the aforementioned biomarkers, higher serum levels of SCF—the c-kit ligand—were associated with less MF, preserved LVEF, and better outcomes in our study cohort, which is consistent with cardioprotective actions via progenitor-cell recruitment, neovascularization, and reduced apoptosis/remodelling.^10^

Given the exploratory, hypothesis-generating nature of our study, these findings need to be validated and expanded in larger, prospective cohorts. Specifically, GH, FGF23 and SCF emerge as novel serum biomarkers in severe AS with potential relevance for more comprehensive risk stratification as they were identified to link MF, cardiac dysfunction and cardiovascular mortality in this pathological condition.

## Authors’ contributions

S.G. conceived the study, performed data acquisition, performed statistical analyses, interpreted data, wrote the manuscript draft and revised it. J.P.F. performed statistical analyses, interpreted data and reviewed and revised the manuscript. A.L. performed statistical analyses, interpreted data and reviewed and revised the manuscript. P.B. performed data acquisition and revised the manuscript. S.v.H. discussed results and strategy, interpreted data and revised the manuscript. A.F. discussed results and strategy, interpreted data and revised the manuscript. BEB performed data acquisition. E.M.Z. performed histological measurements and revised the manuscript. M.P. performed echocardiography. C.S. discussed results and revised the manuscript. K.T. performed TAVR, discussed results and strategy, interpreted data and revised the manuscript. G.H. is the trial’s principal investigator, provided serum samples for biomarker measurements, discussed results and strategy, interpreted data and revised the manuscript. M.S. conceived, designed and directed the study (lead), was involved in all analyses and wrote the manuscript. All authors have read and approved the final manuscript.

## Data Availability

The data underlying this article will be shared on reasonable request to the corresponding author.
